# Blood-based DNA methylation markers for lung cancer prediction

**DOI:** 10.1136/bmjonc-2024-000334

**Published:** 2024-05-30

**Authors:** Justina Ucheojor Onwuka, Florence Guida, Ryan Langdon, Mikael Johansson, Gianluca Severi, Roger L Milne, Pierre-Antoine Dugué, Melissa C Southey, Paolo Vineis, Torkjel Sandanger, Therese Haugdahl Nøst, Marc Chadeau-Hyam, Caroline Relton, Hilary A. Robbins, Matthew Suderman, Mattias Johansson

**Affiliations:** 1 Genomic Epidemiology Branch, International Agency for Research on Cancer, Lyon, France; 2 Population Health Sciences, Bristol Medical School, University of Bristol, Bristol, UK; 3 MRC Integrative Epidemiology Unit, Population Health Sciences, Bristol Medical School, University of Bristol, Bristol, UK; 4 Department of Radiation Sciences Oncology, Umeå University, Umea, Sweden; 5 ‘Exposome, Heredity, Cancer and Health’ Team, Gustave Roussy, Universite Paris-Saclay, Villejuif, Île-de-France, France; 6 Department of Statistics, Computer Science, University of Florence, Firenze, Toscana, Italy; 7 Cancer Epidemiology Division, Cancer Council Victoria, Melbourne, Victoria, Australia; 8 Centre for Epidemiology and Biostatistics, Melbourne School of Population and Global Health, The University of Melbourne, Melbourne, Victoria, Australia; 9 Precision Medicine, School of Clinical Sciences at Monash Health, Monash University, Clayton, Victoria, Australia; 10 Department of Clinical Pathology, Melbourne Medical School, The University of Melbourne, Melbourne, Victoria, Australia; 11 MRC Centre for Environment and Health, School of Public Health, Imperial College London, London, UK; 12 Department of Community Medicine, Faculty of Health Sciences, UiT The Arctic University of Norway, Tromso, Troms, Norway; 13 School of Public Health, Imperial College London, London, UK; 14 MRC Centre for Environment and Health, Imperial College London, London, UK; 15 MRC Integrative Epidemiology Unit, Population Health Sciences, Bristol Medical School, University of Bristol, Bristol, Bristol, UK

**Keywords:** Biomarkers, Lung cancer (non-small cell), Epidemiology, Lung cancer (small-cell)

## Abstract

**Objective:**

Screening high-risk individuals with low-dose CT reduces mortality from lung cancer, but many lung cancers occur in individuals who are not eligible for screening. Risk biomarkers may be useful to refine risk models and improve screening eligibility criteria. We evaluated if blood-based DNA methylation markers can improve a traditional lung cancer prediction model.

**Methods and analysis:**

This study used four prospective cohorts with blood samples collected prior to lung cancer diagnosis. The study was restricted to participants with a history of smoking, and one control was individually matched to each lung cancer case using incidence density sampling by cohort, sex, date of blood collection, age and smoking status. To train a DNA methylation-based risk score, we used participants from Melbourne Collaborative Cohort Study-Australia (n=648) and Northern Sweden Health and Disease Study-Sweden (n=380) based on five selected CpG sites. The risk discriminative performance of the methylation score was subsequently validated in participants from European Investigation into Cancer and Nutrition-Italy (n=267) and Norwegian Women and Cancer-Norway (n=185) and compared with that of the questionnaire-based PLCOm2012 lung cancer risk model.

**Results:**

The area under the receiver operating characteristic curve (AUC) for the PLCOm2012 model in the validation studies was 0.70 (95% CI: 0.65 to 0.75) compared with 0.73 (95% CI: 0.68 to 0.77) for the methylation score model (*P*_difference_=0.07). Incorporating the methylation score with the PLCOm2012 model did not improve the risk discrimination (AUC: 0.73, 95% CI: 0.68 to 0.77, *P*_difference_=0.73).

**Conclusions:**

This study suggests that the methylation-based risk prediction score alone provides similar lung cancer risk-discriminatory performance as the questionnaire-based PLCOm2012 risk model.

**WHAT IS ALREADY KNOWN ON THIS TOPIC**

- Given the high level of sensitivity with which DNA methylation reflects lifelong exposure to tobacco smoke, can a blood-based DNA methylation signature improve risk assessment for lung cancer among individuals with a smoking history, either as a standalone marker or in combination with an existing smoking history-based risk model?

**WHAT THIS STUDY ADDS**

- We trained a DNA methylation-based risk score using pre-diagnostic blood samples from two population cohorts from Australia and Sweden based on five CpG sites. We validated the DNA methylation-based risk score in two separate cohorts from Italy and Norway and compared with that of the questionnaire-based PLCOm2012 lung cancer risk model. We found that methylation-based risk prediction score alone matched or slightly surpassed the traditional lung cancer prediction model (PLCOm2012) in discriminating between future lung cancer cases and controls.

**HOW THIS STUDY MIGHT AFFECT RESEARCH, PRACTICE OR POLICY**

- Since self-reported smoking history may be influenced by recall bias and differences in cigarette smoking behaviour, a methylation-based risk prediction score can replace a traditional questionnaire-based model for personalised lung cancer risk assessment.

## Background

Several randomised trials have demonstrated that screening with low-dose CT (LDCT) is effective in reducing lung cancer mortality.^1–3^ In contrast to screening modalities for other cancers, lung cancer screening is targeted to individuals not only based on age but to those at high risk based on their smoking history. The US Preventive Services Task Force (USPSTF) has recommended LDCT screening for individuals aged 50–80 years who have at least 20 pack-years of smoking exposure, including former smokers who quit less than 15 years ago.^4^ Several countries are now piloting or implementing lung cancer screening. However, many incident lung cancer cases do not meet current screening eligibility criteria, despite having a history of smoking.^5 6^

Eligibility criteria for LDCT screening is currently either based on categorical eligibility criteria such as USPSTF 2021 criteria^4^ or absolute lung cancer risk thresholds calculated using risk-prediction models such as the PLCOm2012 model.^7^ Compared with the 2021 USPSTF criteria, the PLCOm2012 model can enrich the screened population with those at highest risk and most likely to benefit from screening.^8^ Recently, the USPSTF suggested that to improve detection and minimise false positive results from LDCT, there is need for research to identify biomarkers that can more accurately identify high-risk individuals.^4^

DNA methylation, the addition of methyl groups to cytosine residues in genomic DNA, constitutes a potential biomarker for lung cancer risk stratification.^9–11^ Epigenome-wide association studies (EWAS) have identified CpG sites in germline DNA, commonly extracted from whole blood, with methylation levels that are consistently associated with smoking history,^12–15^ as well as risk of lung cancer.^12 16 17^ It is important to distinguish DNA methylation measured in germline DNA to other methylation-based markers based on circulating cell-free DNA. Cell-free DNA methylation changes generally reflect an established cancer,^18–22^ whereas germline DNA methylation may be thought of as an objective marker of smoking exposure history. Given the high level of sensitivity with which DNA methylation reflects lifelong exposure to tobacco smoke, this may provide opportunities to replace or improve on risk models based on self-reported tobacco-exposure history. In a previous study, Battram *et al* identified 16 CpG sites that were associated with risk of lung cancer, 14 of which had been associated with smoking.^23^

The current study aimed to evaluate if it is possible to define a blood-based DNA methylation signature that improves risk assessment for lung cancer among individuals with a smoking history, either as a standalone marker or in combination with an established smoking history-based risk model.

## Methods

### Study design and sample

To assess whether a blood-based DNA methylation panel can outperform or improve a standard questionnaire-based lung cancer prediction model in identifying individuals for lung cancer screening, we repurposed the four pre-diagnostic data sets from previous EWAS in whole blood by Battram *et al*.^23^ These included the Melbourne Collaborative Cohort Study (MCCS), the Northern Sweden Health and Disease Study (NSHDS), the European Investigation into Cancer and Nutrition (EPIC-Italy) and the Norwegian Women and Cancer (NOWAC). We trained a methylation score in two of the cohorts based on the 16 CpG sites identified by Battram *et al* as robustly associated with the risk of lung cancer. We subsequently tested the risk-discriminative performance of the methylation score in the remaining two cohorts and compared it to the extensively validated PLCOm2012 risk-prediction model.^7^

In this study, we combined the cohorts into training and validation sets based on their similarity in matching factors. Specifically, we used MCCS (324, case-control pairs) and NSHDS (190, case-control pairs) as the training set (smoking-matched) and EPIC-Italy (n=160 cases and n=107 controls) and NOWAC (n=115 cases and n=70 controls) as the validation set (not smoking-matched). For all cohorts, participants were free of cancer at enrolment and lung cancer cases were defined as all invasive cancers coded C34.0 to C34.9 in the International Classification of Diseases for Oncology, Third Edition.^24^ For each case, one control was selected as follows:

#### Training set

For each case, one control subject was matched by cohort, sex, date of blood collection (within 6 months), date of birth (within 1 year) and smoking status in five categories; never smokers, former smokers (<10 years, ≥10 years since quitting) and current smokers (<15 or ≥15 cigarettes per day).

#### Validation set

In EPIC-Italy, healthy controls were individually matched to incident cases by sex, date of birth (±5 years), date of inclusion in the study and study centre. In the NOWAC cohort, one control with an available blood sample was selected per case, matched on time since blood sampling and year of birth respectively.

### DNA methylation assays

The DNA methylation measurement and data pre-processing for MCCS, NSHDS, EPIC-Italy and NOWAC have been described previously.^25^ Briefly, genome-wide DNA methylation analyses were performed on pre-diagnostic blood samples using the Illumina Infinium Human Methylation 450 K array. The samples from NOWAC and EPIC-Italy were assayed at the Human Genetics Foundation (Turin, Italy), whereas the samples from MCCS and NSHDS were assayed at the Universities of Melbourne (Australia) and Bristol (UK), respectively. For each CpG site considered, we used the methylation levels represented by beta values ranging from 0 to 1.

### Statistical analysis

#### Missing data imputation

We imputed missing information for Body Mass Index (BMI), education, years smoked and cigarettes smoked per day. For the latter three variables, which were partially missing, we stratified by cohort and smoking status (current or former) and applied multivariate imputation by chained equations with age, gender, case-control status, cigarettes smoked per day, years smoked, quit years for former smokers and education as predictors. We imputed the mean value for BMI, which was missing in about 0.3% of participants. In NSHDS (Sweden), information on cigarettes smoked per day was missing for both former and current smokers. We used EPIC-Italy as reference to impute cigarettes smoked per day in NSHDS. We imputed missing values as mean values for 5 CpGs for which methylation data were missing in less than 0.5% of the participants. We did not conduct imputation for race or ethnicity, chronic obstructive pulmonary disease and family history of lung cancer variables since no information was available for these variables.

#### DNA methylation levels and smoking history

The association between smoking history and DNA methylation levels was assessed using linear regression. These analyses were adjusted for age, sex, case-control status, and cohort.

#### Questionnaire-based risk estimation for study participants

We estimated the risk for each included study participant based on the PLCOm2012 model. The predictor variables included in the PLCOm2012 model are age, education, BMI, family history of lung cancer and smoking status (current and former), smoking intensity, smoking duration and time since quitting in former smokers.^7^

#### DNA methylation-based prediction panel

To identify a panel of stable risk-informative CpG sites among the 16 reported in Battram *et al*,^23^ we partitioned the training set (MCCS and NSHDS) into 500 random splits at a 3:1 ratio of case-control pairs. We then applied least absolute shrinkage and selection operator (LASSO) logistic regression on each of the larger splits while adjusting for the matching factors. We used the smaller split for predicting the fitted models. Suitable shrinkage parameters (λ) were identified through 10-fold cross-validation. The final panel of risk-informative CpG sites was identified as those selected by the LASSO logistic regression with a frequency of at least 80% (at least 400 of the 500 splits) for subsequent risk modelling.

The final risk scores were fitted in the full training set using (unpenalised) unconditional logistic regression models with adjustment for the matching factors (age, sex, smoking status in four categories). Each score included the selected set of CpG sites, and either with (β1×cg05575921 + β2×cg06126421 + β3×cg21566642 + β4×cg23387569 + β5×cg26963277 + β6×logit (PLCOm2012)) or without (β1×cg05575921+ β2×cg06126421+ β3×cg21566642 + β4×cg23387569 + β5×cg26963277) the logit of the PLCOm2012 model-based risk estimates.

#### Discrimination analyses

We used receiver operating characteristic (ROC) curves to evaluate the extent to which the methylation score could discriminate between lung cancer cases and controls using the validation set (EPIC-Italy and NOWAC). We estimated the area under the curve (AUC) for three risk scores while adjusting for the matching factors (age and sex) and smoking status in four categories: (a) methylation score alone (AUC_methscore_); (b) PLCOm2012 model alone (AUC_PLCOm2012_) and (c) an integrated score based on the methylation score combined with PLCOm2012 model (AUC_integrated_). We subsequently conducted stratified discrimination analyses by age, sex, smoking status, lead time, eligibility by USPSTF screening criteria or PLCOm2012-based risk thresholds, and cohort.

All statistical analyses were carried out using R V.4.0.4. The LASSO was performed using glmnet. The ROC curves were plotted with R package pROC.

#### Patient and public involvement

Patients or the public were not involved in the design, or conduct, or reporting, or dissemination plans of our research.

#### Sensitivity analyses

We also trained a methylation score using the MCCS cohort alone—without the NSHDS cohort in training set where cigarettes per day were imputed—and validated the resulting models using the same approach and validation set as in the main analysis.

## Results

### Baseline characteristics

Of the 1799 participants in the four combined cohorts (online supplemental table 1), we included a total of 1480 participants who ever smoked in our risk prediction analysis (table 1). The final training set (MCCS and NSHDS) included 514 case-control pairs, and the final validation set (EPIC-Italy and NOWAC) included 275 cases and 177 controls. The cases and controls in the validation cohorts were majority female (64/61% vs 39/39% in the training set), and younger (mean (SD): 55.4 (5.4) vs 57.7 (7.1) years in the training set).

10.1136/bmjonc-2024-000334.supp1 Supplementary data

**Table 1:** Characteristics of formerly/currently smoking participants in the combined dataset from four cohorts (NSHDS, MCCS, EPIC and NOWAC)

	Training set		Validation set	
Characteristic	Case, n=514	Control, n=514	Case, n=275	Control, n=177
Gender, N (%)				
Male	312 (61%)	312 (61%)	99 (36%)	69 (39%)
Female	202 (39%)	202 (39%)	176 (64%)	108 (61%)
Age, mean (SD)	57.7 (7.1)	57.6 (7.1)	55.4 (5.6)	55.0 (5.4)
BMI, mean (SD)	26.4 (4.0)	27.1 (4.4)	25.5 (4.2)	25.5 (3.6)
Missing	0	2	2	0
Pre-diagnosis lead time, mean (SD)	9.3 (4.9)		5.7 (3.5)	
Missing	0	514	0	177
Cohort, N (%)				
MCCS	324 (63%)	324 (63%)		
NSHDS	190 (37%)	190 (37%)		
EPIC			160 (58%)	107 (60%)
NOWAC			115 (42%)	70 (40%)
Smoking status, N (%)				
Current	287 (56%)	287 (56%)	182 (66%)	78 (44%)
Former	227 (44%)	227 (44%)	93 (34%)	99 (56%)
Years smoked, mean (SD)	35.9 (10.3)	33.6 (12.0)	33.3 (10.2)	27.4 (11.2)
Missing			2	3
Average number of cigarettes smoked per day, mean (SD)	22.7 (13.8)	19.6 (13.2)	14.6 (7.2)	11.7 (7.4)
Missing	56	48	2	4
Quit years, mean (SD)	4.3 (7.4)	5.7 (9.7)	3.6 (7.1)	8.1 (10.2)
Eligible by USPSTF 2021 criteria, N (%)	287 (56%)	264 (51%)	133 (48%)	41 (23%)
Eligible for lung cancer screening (PLCOm2012, cut-off: 1.00%), N (%)	219 (48%)	185 (40%)	72 (27%)	23 (14%)
Missing	56	50	8	7
6-year risk by PLCOm2012, mean (SD)	1.3% (1.2%)	1.1% (1.2%)	0.8% (0.7%)	0.4% (0.5%)
Missing			8	7

BMI, Body Mass Index; EPIC, European Investigation into Cancer and Nutrition; MCCS, Melbourne Collaborative Cohort Study; NOWAC, Norwegian Women and Cancer; NSHDS, Northern Sweden Health and Disease Study; USPSTF, US Preventive Services Task Force.

Based on the USPSTF 2021 lung cancer screening criteria, 56% of cases and 51% of controls in the training cohorts, and 48% of cases and 23% of controls in the validation cohorts, were eligible for screening^4^ (table 1). The mean 6-year risk of developing lung cancer was estimated using the PLCOm2012 model as 1.3% for cases and 1.1% for controls in the training set, and 0.8% for cases and 0.4% for controls in the validation set (table 1). The baseline characteristics of lung cancer cases who did not meet the USPSTF2021 screening criteria are reported in online supplemental table 3, with corresponding information on controls in online supplemental table 4.

### Association between smoking history and DNA methylation levels

We first evaluated the 16 risk CpG sites identified by Battram *et al* 23 in relation to different smoking parameters using the complete data set (online supplemental table 1). Current smoking participants had significantly lower DNA methylation levels in all the CpG sites compared with never smokers (*ptrend* <0.05) (online supplemental figure 1). Former smoking participants who smoked more than 30 cigarettes per day and those who smoked for at least 30 years also had lower DNA methylation levels than never smokers in most of the CpG sites (*ptrend* <0.05) (online supplemental figures 2 and 3).

### Training of methylation-based risk-score

**Figure 1:**
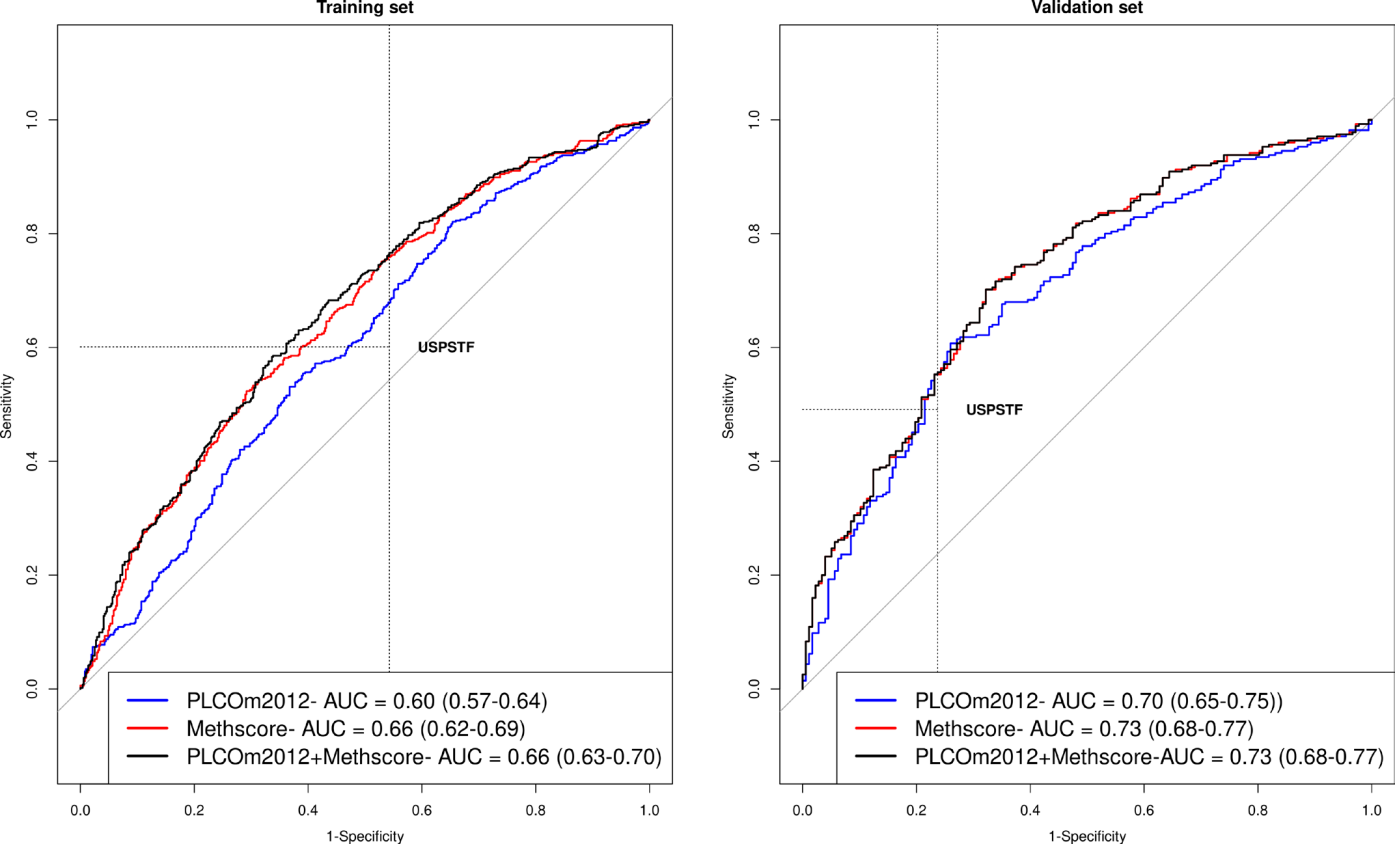
Risk-discriminatory performance depicted using ROC curves for three prediction models in the training and validation set, including the PLCOm2012 risk model, the methylation risk score (methscore) and the integrated PLCOm2012+methscore. ROC, receiver operating characteristic.

Of the 16 risk CpG sites that were considered (online supplemental table 2), 5 were selected to be included in the final methylation risk score (selected in at least 80% of 500 re-samplings using LASSO) (online supplemental figure 4). These CpG sites were cg21566642 (ALPPL2), cg23387569 (AGAP2), cg06126421 (IER3), cg05575921 (AHRR) and cg26963277 (KCNQ1OT1). Table 2 shows β-coefficients for three risk scores that were fitted in the full training set: the PLCOm2012 model alone, the methylation markers alone and the PLCOm2012 combined with the methylation markers. The apparent AUCs in the training set were 0.60 (95% CI: 0.57 to 0.64) for the PLCOm2012, 0.66 (95% CI: 0.62 to 0.69) for the methylation score and 0.66 (95% CI: 0.63 to 070) for the integrated risk score (figure 1).

**Table 2:** β-coefficients and multivariable ORs with 95% CIs for lung cancer risk factors in the training set

	CHR	Gene name	PLCOm2012 model		Methylation risk score (MRS)		PLCOm2012+MRS	
			β	OR (95% CI)	β	OR (95% CI)	β	OR (95% CI)
PLCO 2012 logit			0.14	1.15 (1.06 to 1.27)			0.13	1.13 (1.04 to 1.24)
cg05575921, per SD	5	AHRR			−0.17	0.85 (0.66 to 1.08)	−0.16	0.85 (0.67 to 1.08)
cg06126421, per SD	6	IER3			−0.18	0.83 (0.71 to 0.98)	−0.17	0.84 (0.71 to 1.00)
cg21566642, per SD	2	ALPPL2			−0.31	0.73 (0.60 to 0.89)	−0.30	0.74 (0.61 to 0.91)
cg23387569, per SD	12	AGAP2			−0.21	0.81 (0.71 to 0.93)	−0.22	0.80 (0.70 to 0.92)
cg26963277, per SD	11	KCNQ1OT1			−0.06	0.94 (0.82 to 1.09)	−0.07	0.93 (0.81 to 1.08)

Models were adjusted for matching factors: cohort, sex, age and smoking status (former smokers with <10 or ≥10 years since quitting, and current smokers with <15 or ≥15 cigarettes smoked per day).

### Risk discriminative performance in the validation set

In the validation set, the overall AUC for the PLCOm2012 score was 0.70 (95% CI: 0.65 to 0.75) compared with 0.73 (95% CI: 0.68 to 0.77) for the methylation score (AUC_PLCOm2012_ vs AUC_methscore_, *p-difference*=0.07) (figure 1). The AUC for the integrated risk score based on both the PLCOm2012 model and the methylation score was 0.73 (AUC_integrated_, 95% CI: 0.68 to 0.77, AUC_integrated_ vs AUC_methscore_, *p-difference*=0.73 and *p-difference*=0.08 for difference in AUC with PLCOm2012 alone) (figure 1). The AUC estimates were similar for the methylation score and integrated models in most strata (table 3). We note that the AUC estimates in the validation set were higher than in the training set, and the reason for this is that the controls in the training sample were matched by smoking status to the index cases. Smoking-matched design accounts for the risk discrimination afforded by smoking status.

**Table 3:** Risk-discriminatory performance estimated using AUC for three prediction models in the validation set, including the PLCOm2012 risk model, the methylation risk score (methscore) and the integrated PLCOm2012+methscore

	Number of cases	Number of controls	PLCOm2012 model AUC (95% CI)	P value	Methylation risk score (methscore) AUC (95% CI)	P value	PLCOm2012+methscore AUC (95% CI)	P value
Overall	275	177	0.70 (0.65 to 0.75)		0.73 (0.68 to 0.77)		0.73 (0.68 to 0.78)	
Age								
<60	209	141	0.69 (0.63 to 0.74)	0.68	0.72 (0.66 to 0.77)	0.48	0.72 (0.66 to 0.77)	0.49
≥60	66	36	0.71 (0.61 to 0.82)		0.76 (0.65 to 0.86)		0.76 (0.65 to 0.86)	
Sex								
Male	99	69	0.72 (0.64 to 0.8)	0.58	0.73 (0.66 to 0.81)	0.79	0.73 (0.66 to 0.81)	0.78
Female	176	108	0.69 (0.63 to 0.76)		0.72 (0.66 to 0.78)		0.72 (0.66 to 0.78)	
Smoking status								
Current	182	78	0.67 (0.6 to 0.74)	0.32	0.71 (0.64 to 0.78)	0.38	0.71 (0.64 to 0.78)	0.38
Former	93	99	0.61 (0.53 to 0.69)		0.66 (0.58 to 0.74)		0.66 (0.58 to 0.74)	
Pre-diagnosed lead time cases and all controls, years								
<5.3	139	177	0.70 (0.64 to 0.76)	0.97	0.73 (0.67 to 0.79)	0.89	0.73 (0.67 to 0.79)	0.88
≥5.3	136	177	0.70 (0.64 to 0.76)		0.72 (0.67 to 0.78)		0.72 (0.67 to 0.78)	
USPSTF 2021								
Yes	135	42	0.65 (0.55 to 0.74)	0.85	0.64 (0.55 to 0.73)	0.13	0.64 (0.55 to 0.73)	0.12
No	140	135	0.66 (0.59 to 0.72)		0.72 (0.66 to 0.78)		0.72 (0.66 to 0.78)	
PLCOm2012 (threshold: 1.00%)								
Yes	73	24	0.66 (0.52 to 0.80)	0.80	0.72 (0.59 to 0.84)	0.95	0.72 (0.59 to 0.84)	0.94
No	202	153	0.68 (0.62 to 0.73)		0.71 (0.66 to 0.77)		0.71 (0.66 to 0.77)	
Cohort								
EPIC-Italy	160	107	0.73 (0.67 to 0.79)	0.79	0.76 (0.70 to 0.82)	0.11	0.76 (0.70 to 0.82)	0.12
NOWAC	115	70	0.65 (0.56 to 0.73)		0.68 (0.60 to 0.76)		0.68 (0.60 to 0.76)	

Models adjusted for age, and sex (matching factors) and smoking status in four categories.

AUC, area under the curve; EPIC, European Investigation into Cancer and Nutrition; NOWAC, Norwegian Women and Cancer; USPSTF, US Preventive Services Task Force.

### Sensitivity analyses

When using the MCCS cohort only as training set, four CpG sites (cg21566642, cg23387569, cg06126421 and cg25305703) (online supplemental figure 5) were selected to be included in the methylation risk score (selected in at least 80% of 500 re-samplings). Of these four CpG sites, three (cg21566642, cg23387569 and cg06126421) were common to those selected in the main analysis. In the validated set, the AUC for this methylation score and corresponding integrated risk score (online supplemental table 5) was similar to those of the main analysis (online supplemental figure 6).

## Discussion

We developed and validated a methylation-based risk score measured in pre-diagnostic blood DNA and compared its performance with that of an established traditional lung cancer risk model in study participants with a history of regular smoking exposure. We found that a methylation-based risk score with five CpG sites matched or slightly surpassed the PLCOm2012 model in discriminating between future lung cancer cases and controls. Combining the PLCOm2012 model and methylation markers did not further improve risk discrimination.

Screening high-risk individuals with a history of smoking exposure reduces lung cancer mortality.^1^ However, accurately identifying high-risk individuals as screening-eligible remains a challenge. The PLCOm2012 model predicts lung cancer risk better than the USPSTF2021^8^ but uses self-reported smoking history, which may be influenced by recall bias and differences in cigarette smoking behaviour.^26^ Biomarkers, such as cotinine and certain DNA methylation sites/markers, may provide more objective measures of tobacco exposure. Cotinine is a marker of short-term smoking exposure^27^ whereas DNA methylation markers can inform on long-term smoking exposure.^28^

Environmental exposures can alter epigenetic patterns, and thereby stably influence gene expression, without changing the nucleotide sequence across these cell divisions, often resulting in changes in phenotype-persistent changes to molecular phenotypes.^29^ There are a series of published studies reporting extensive changes to DNA methylation associated with biological reflection/signature/imprint of smoking exposure to cigarette smoke, including a meta-analysis which identified differences in over 2600 CpG sites between smokers and never smokers.^30^ Smoking remains the most pronounced determinants of DNA methylation variation studied to date. Its impact is so marked that its effect is detected in epigenome-wide association studies of smoking-related outcomes, hence the observation that smoking-related changes predominate in EWAS of lung cancer.^31^ Because DNA methylation reflects biological smoking exposure, and its effect attenuates over time, it is a conceptually attractive candidate for risk stratification in both individuals who actively smoke and in individuals who have quit smoking.

Bojesen *et al* demonstrated that the AHRR (cg05575921) methylation alone performed similarly to the PLCOm2012 model in predicting lung cancer risk among participants who smoked. The current study confirms this finding^32^: using 514 case-control pairs, we developed a methylation-based risk score using five CpG sites that was validated in two external cohorts of 275 cases and 177 controls. We found that our methylation risk score alone slightly outperformed PLCOm2012 model in most relevant strata. Combining the methylation score with the PLCOm2012 model did not improve risk discrimination further. This suggests that the majority of lung cancer risk information contained among the selected CpG sites come from their ability to represent tobacco exposure history. Of the five CpG sites included in our methylation risk score, cg05575921 (AHRR) is the most well-established biomarker of smoking exposure.^32–34^ A study by Jacobsen *et al* suggested that integrating cg05575921 (AHRR) methylation with NLST screening criteria can improve the specificity of lung cancer screening by excluding those individuals with the lowest lung cancer risk from the eligible population.^35^

Given the wealth of additional informative smoking-associated methylation sites that have been reported, including those relevant to different ethnic groups,^36 37^ there is high potential to improve the five CpG site score defined in this study. Differential DNA methylation patterns have also been identified in studies of never smoking lung cancer cases.^38^ This observation raises the further possibility of extending a DNA methylation score beyond capturing smoking-related variation. A more comprehensive analysis of the use of much higher numbers of informative CpG sites on additional prospective cohort studies is warranted to enhance the discriminatory performance of a methylation score-based model.

One of the key strengths of our study is its prospective and population-based design, and most importantly the use of pre-diagnostic blood DNA. This study design minimises the possibility that the CpG sites studied are affected by the presence of an undetected developing tumour for most cases included in our study. Second, our approach involved training and testing of the methylation risk score in independent cohorts, a crucial and unique strength of our study. We also had a sufficient sample size to identify any meaningful differences in risk discrimination between standard and methylation-based risk scores. A potential limitation of our study is that both the training and validation cohorts were included in the original EWAS that identified the CpG sites taken forward for use in the prediction model. Whereas this may in theory result in some optimism in the risk discriminative performance of the methylation score in our validation sample,^39^ such bias is likely to be minimal because only the training cohorts were used to estimate the CpG site-specific parameters effect used in the methylation score. Another limitation of our study is the homogeneous nature of the included cohorts with predominantly white study participants. Future studies with diversity in race and ethnicity are therefore warranted to evaluate the transportability of methylation markers as lung cancer risk indicators. Importantly, because we used matched case-control studies, the AUC estimates do not reflect the performance that would have been seen in a random sample because the risk-discriminative performance afforded by age and sex (as well as smoking status in the training sample) has already been accounted for. Although this implies that the magnitude of the AUCs would differ in a random population sample, comparing the risk discriminative performance of different models is still valid using this design. We also note that our study design does not readily allow us to establish an absolute risk model, which is a pre-requisite for translation into a practical screening situation. Future studies should therefore be conducted using a design that facilitates the development of absolute risk models, such as case-cohort or full cohort analysis.

A major challenge for current screening programmes is that approximately half of incident lung cancer cases are not eligible for LDCT. It is well-established that risk stratification can improve the effectiveness of lung cancer screening programmes by identifying more future cases without screening more people, but few screening programmes have implemented individualised risk assessment prior to screening. Risk biomarkers have the potential to further improve risk assessment. In reflecting on the implications of our findings, and those of previous studies, it is not yet clear that a risk score based on smoking-associated CpG sites can provide important improvements in risk discrimination over and above that afforded by traditional questionnaire-based risk models. Rather, germline DNA methylation markers may be useful as a complementary means for risk assessment in situations where accurate smoking history is challenging to attain. Such molecular markers may provide patients and physicians with an objective measure of individualised risk for personalised decision making to reduce harm and improve benefits of screening. It is also possible that objective risk biomarkers—such as a methylation risk score—may circumvent the potential stigma associated with smoking in risk assessment, thereby motivating more individuals at risk to engage in lung cancer screening programmes. It will also be important to evaluate this hypothesis in a carefully designed study that evaluates acceptability of biomarker-based risk assessment in participants representative of the target population.

## Conclusion

Our study indicates that a smoking-related lung cancer risk model based on five germline CpG sites can replace a traditional questionnaire-based model for personalised lung cancer risk assessment but does not provide important improvements in risk discrimination to that of a traditional questionnaire-based risk model. Larger panels of CpG sites should be explored in population-representative samples to enhance future models of this type.

## Data Availability

Data are available upon reasonable request. The datasets analysed are not publicly available for confidentiality reasons. However, the corresponding author may make the anonymized version available upon request.
