# Supplementary material for: Blood-based DNA methylation markers for lung cancer prediction
Source: BMJ Oncol. 2024 May 30;3(1):e000334. doi: 10.1136/bmjonc-2024-000334 (PMC11234992; doi:10.1136/bmjonc-2024-000334)

Blood-based DNA methylation markers for lung cancer prediction

Justina Ucheojor Onwuka[1], Florence Guida[2], Ryan Langdon[3,14], Mikael Johansson[4], Gianluca Severi[5], Roger L Milne[6,7,8], Pierre-Antoine Dugué[6,7,8], Melissa C. Southey[6,8,9], Paolo Vineis[10], Torkjel Manning Sandanger[11], Therese Haugdahl Nøst[11], Marc Chadeau-Hyam[12,13], Caroline Relton[14], Hilary A. Robbins[1], Matthew Suderman[14], Mattias Johansson[1]

Supplementary Table 1. Characteristics of participants in the combined dataset from 4 cohorts (NSHDS, MCCS, EPIC, and NOWAC).

Characteristic	Case, N = 898	Control, N = 901
**Gender, N (%)**		
Female	455 (51%)	456 (51%)
Male	443 (49%)	445 (49%)
**Age, mean (SD)**	56.7 (6.7)	56.6 (6.8)
**BMI, mean (SD)**	26.1 (4.1)	26.7 (4.3)
Unknown	3	2
**Mean Pre-diagnosis lead time (SD)**	8.2 (4.8)	NA (NA)
Unknown	0	901
**Cohort, N (%)**		
EPIC	186 (21%)	191 (21%)
MCCS	367 (41%)	367 (41%)
NOWAC	129 (14%)	127 (14%)
NSHDS	216 (24%)	216 (24%)
**Smoking status, N (%)**		
Current	469 (52%)	365 (41%)
Former	320 (36%)	326 (36%)
Never	109 (12%)	210 (23%)

Supplementary Table 2. 16 CpG-sites associated with lung cancer risk

CpGs	CHR	Gene Name
cg01901332	11	ARRB1
cg01940273	2	ALPPL2
cg03636183	19	F2RL3
cg05575921	5	AHRR
cg05951221	2	ALPPL2
cg06126421	6	IER3
cg08709672	1	AVPR1B
cg09935388	1	GFI1
cg11660018	11	PRSS23
cg16823042	12	AGAP2
cg21566642	2	ALPPL2
cg23387569	12	AGAP2
cg23771366	11	PRSS23
cg25305703	8	CASC21
cg26963277	11	KCNQ1OT1
cg27241845	2	ALPPL2

Supplementary Table 3. Characteristics of lung cancer cases who do not meet the guidelines of both the USPSTF 2021 and the PLCOm2012 by age.

	Non-eligible, USPTF 2021 criteria			PLCOm2012-based risk≤1.00%		
	Overall	Age <50	Age ≥50	Overall	Age <50	Age ≥50
**Development set**	N = 227	N = 82	N = 145	N = 239	N = 68	N = 171
**Current smoking, N (%)**	95 (42%)	51 (62%)	44 (30%)	147 (62%)	48 (71%)	99 (58%)
**Pack years, N (%)**						
≥20	67 (39%)	48 (71%)	19 (18%)	135 (56%)	48 (71%)	87 (51%)
<20	104 (61%)	20 (29%)	84 (82%)	104 (44%)	20 (29%)	84 (49%)
Unknown	56	14	42			
**Formerly smoked, N (%)**						
≥ 15		8 (9.8%)	51 (35%)	36 (15%)	4 (5.9%)	32 (19%)
< 15		23 (28%)	50 (34%)	56 (23%)	16 (24%)	40 (23%)
**Validation set**	N = 142	N = 102	N = 40	N = 195	N = 156	N = 39
**Current smoking, N (%)**	89 (63%)	62 (61%)	27 (68%)	119 (61%)	93 (60%)	26 (67%)
**Pack years, N (%)**						
≥20	22 (16%)	3 (3.0%)	19 (48%)	86 (44%)	67 (43%)	19 (49%)
<20	118 (84%)	97 (97%)	21 (52%)	109 (56%)	89 (57%)	20 (51%)
Unknown	2	2	0			
**Formerly smoked, N (%)**						
≥ 15	23 (16%)	18 (18%)	5 (12%)	23 (12%)	18 (12%)	5 (13%)
< 15	30 (21%)	22 (22%)	8 (20%)	53 (27%)	45 (29%)	8 (21%)

Supplementary Table 4. Characteristics of controls who do not meet the guidelines of both the USPSTF 2021 and the PLCOm2012 by age.

	Non-eligible, USPTF 2020 criteria			PLCOm2012-based risk≤1.00%		
	Overall	Age <50	Age ≥50	Overall	Age <50	Age ≥50
**Development set**	N = 250	N = 82	N = 168	N = 279	N = 69	N = 210
**Current smoking, N (%)**	97 (39%)	50 (61%)	47 (28%)	161 (58%)	46 (67%)	115 (55%)
**Pack years, N (%)**						
≥20	56 (28%)	41 (56%)	15 (12%)	135 (48%)	38 (55%)	97 (46%)
<20	146 (72%)	32 (44%)	114 (88%)	144 (52%)	31 (45%)	113 (54%)
Unknown	48	9	39			
**Formerly smoked, N (%)**						
≥ 15	79 (32%)	10 (12%)	69 (41%)	58 (21%)	7 (10%)	51 (24%)
< 15	74 (30%)	22 (27%)	52 (31%)	60 (22%)	16 (23%)	44 (21%)
**Validation set**	N = 136	N = 31	N = 105	N = 147	N = 31	N = 116
**Current smoking, N (%)**	58 (43%)	21 (68%)	37 (35%)	61 (41%)	21 (68%)	40 (34%)
**Pack years, N (%)**						
≥20	15 (11%)	9 (29%)	6 (5.9%)	36 (24%)	9 (29%)	27 (23%)
<20	117 (89%)	22 (71%)	95 (94%)	111 (76%)	22 (71%)	89 (77%)
Unknown	4	0	4			
**Formerly smoked, N (%)**						
≥ 15	41 (30%)	3 (9.7%)	38 (36%)	40 (27%)	3 (9.7%)	37 (32%)
< 15	37 (27%)	7 (23%)	30 (29%)	46 (31%)	7 (23%)	39 (34%)

Supplementary Table 5. β-coefficients and Multivariable Odds ratios (OR) with 95% Confidence Intervals (CI) for Lung Cancer Risk Factors in the Training Set (MCCS cohort only)

	CHR	Gene Name	PLCOm2012 model		Methylation risk-score (MRS)		PLCOm2012 + MRS	
			β	OR (95% CI)	β	OR (95% CI)	β	OR (95% CI)
PLCO 2012 logit			0.43	1.53 (1.30-1.83)				
cg06126421, per SD	6	IER3			-0.19	0.82 (0.68-1.00)	-0.17	0.85 (0.70-1.03)
cg21566642, per SD	2	ALPPL2			-0.27	0.76 (0.62-0.94)	-0.22	0.81 (0.65-1.00)
cg23387569, per SD	12	AGAP2			-0.29	0.75 (0.63-0.89)	-0.33	0.72 (0.60-0.86)
cg25305703, per SD	8	CASC21			-0.09	0.91 (0.76-1.09)	-0.12	0.89 (0.74-1.06)

Models were adjusted for matching factors: cohort, sex, age, and smoking status (former smokers with <10 or ≥10 years since quitting, and current smokers with <15 or ≥15 cigarettes smoked per day)

Supplementary Figure 1. Boxplots showing a comparison of DNA methylation levels at 16 CpG-sites between never, former, and current smokers, the combined dataset from 4 cohorts (NSHDS, MCCS, EPIC, and NOWAC). *The *ptrend* from linear regression.

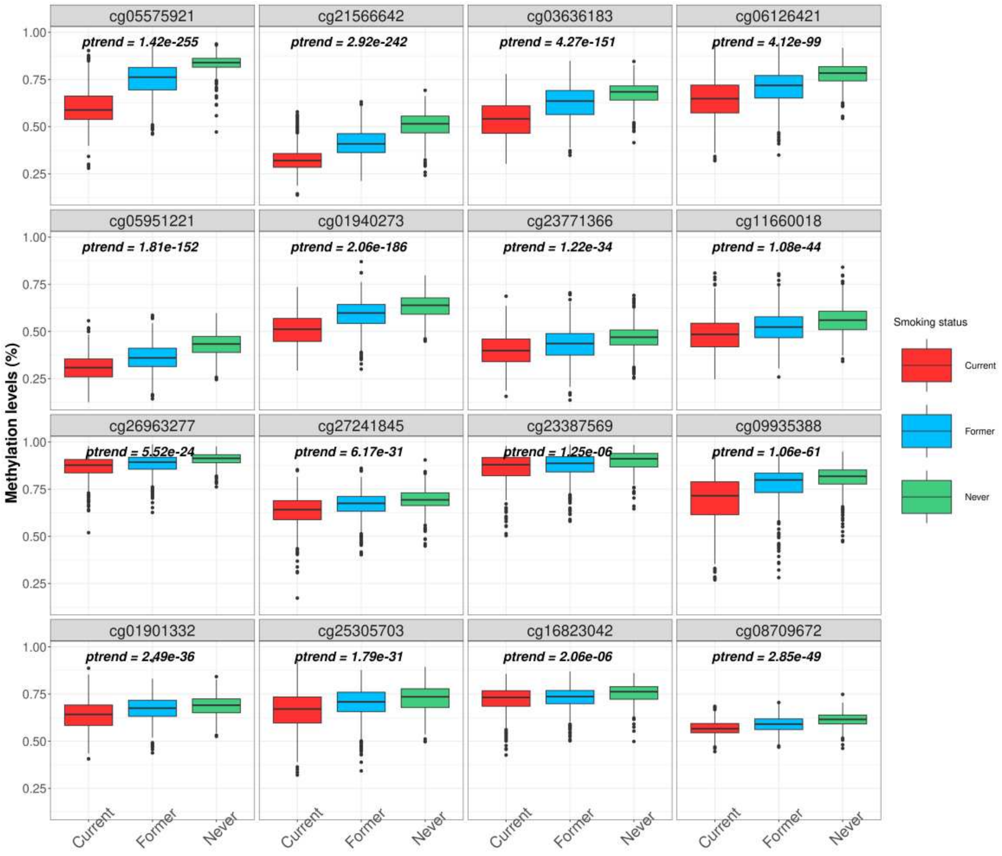

Supplementary Figure 2. Boxplots showing a comparison of DNA methylation levels at 16 CpG-sites between categories of cigarettes per day among currently and formerly smoking participants in the combined dataset from 3 cohorts (MCCS, EPIC, and NOWAC). *The *ptrend* from linear regression.

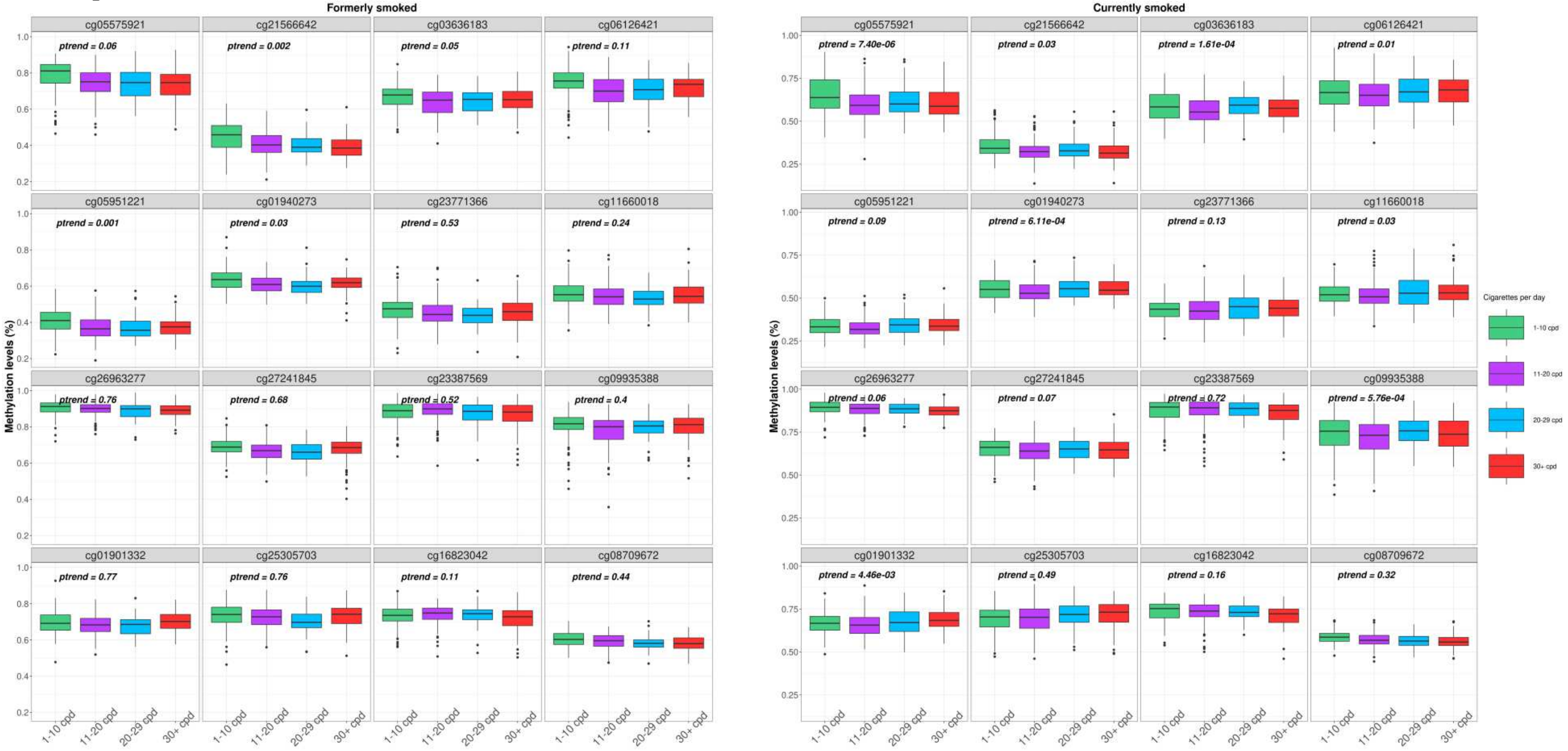

Supplementary Figure 3. Boxplots showing a comparison of DNA methylation levels at 16 CpG-sites between categories of years smoked among currently and formerly smoking participants in the combined dataset from 4 cohorts (NSHDS, MCCS, EPIC, and NOWAC. *The *ptrend* from linear regression.

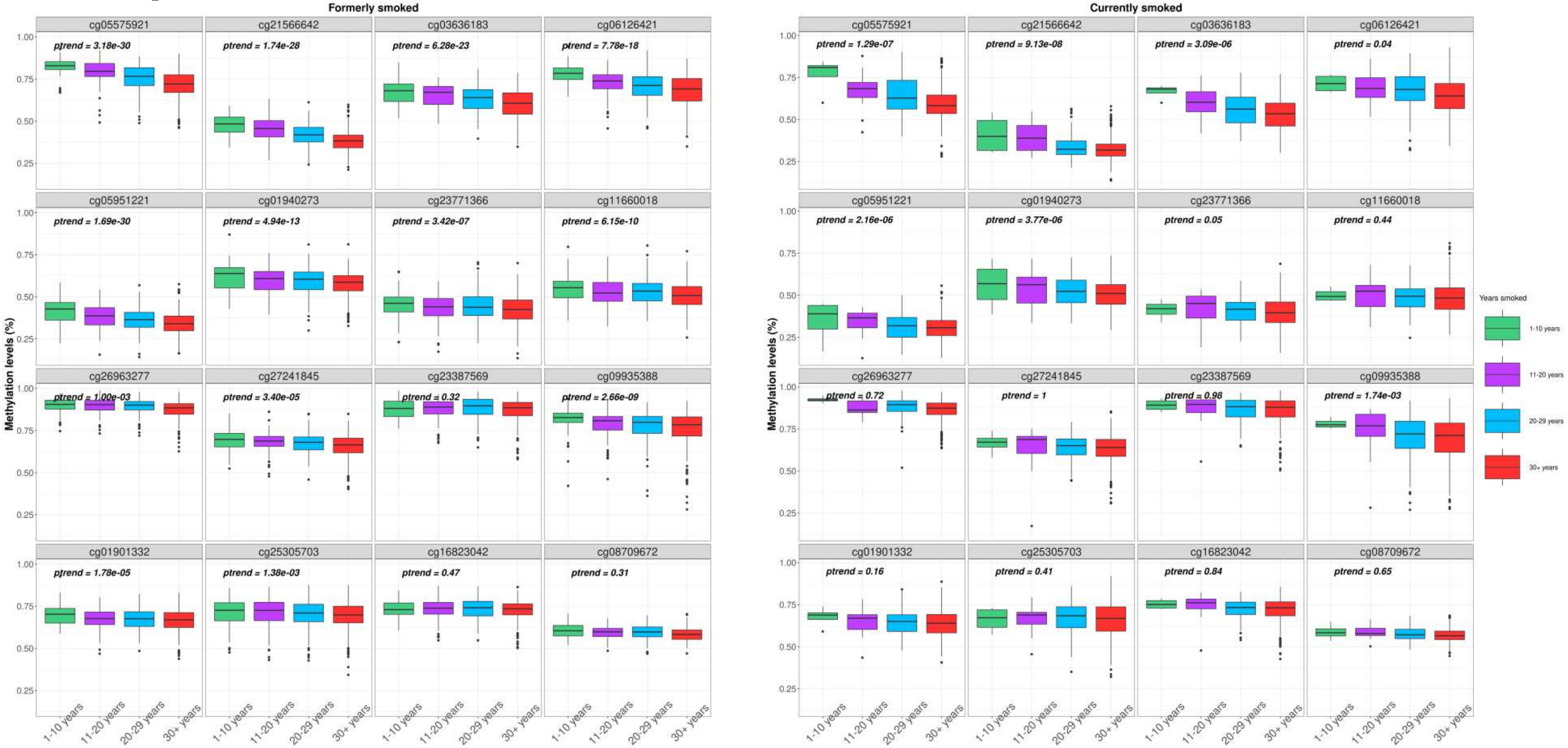

Supplementary Figure 4. Proportion of CpGs selected in 500 different training datasets by LASSO logistic regression model. CpGs selected more than 400 times are marked as black.

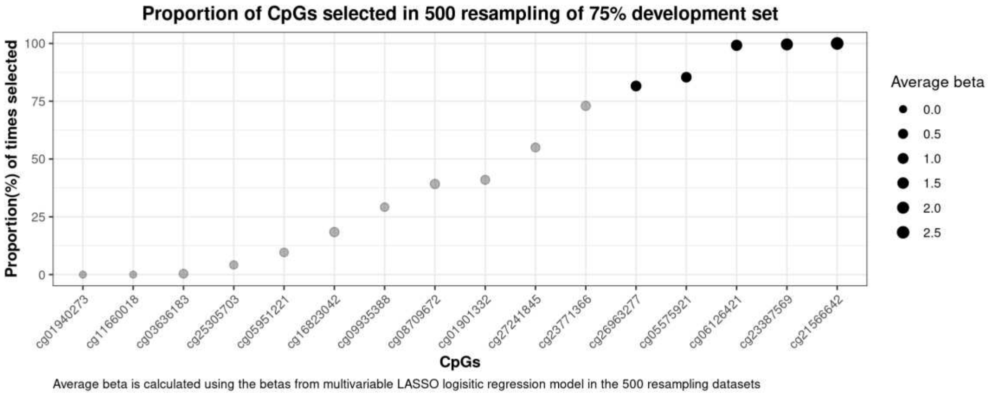

Supplementary Figure 5. Proportion of CpGs selected in 500 different training (MCCS cohort only) sets by LASSO logistic regression model. CpGs selected more than 400 times are marked as black.

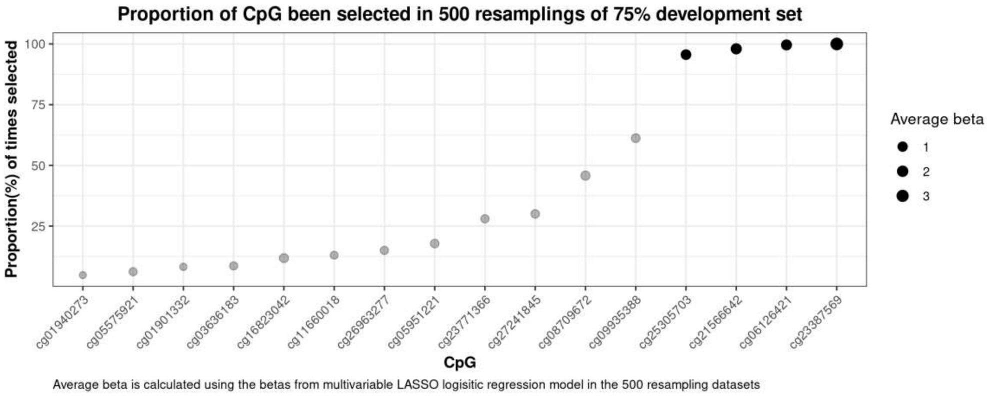

Supplementary Figure 6. Risk-discriminatory performance depicted using ROC-curves for three prediction models in the training (MCCS cohort only) and validation set, including the PLCOm2012 risk-model, the methylation risk-score (methscore), and the integrated PLCOm2012 + methscore.

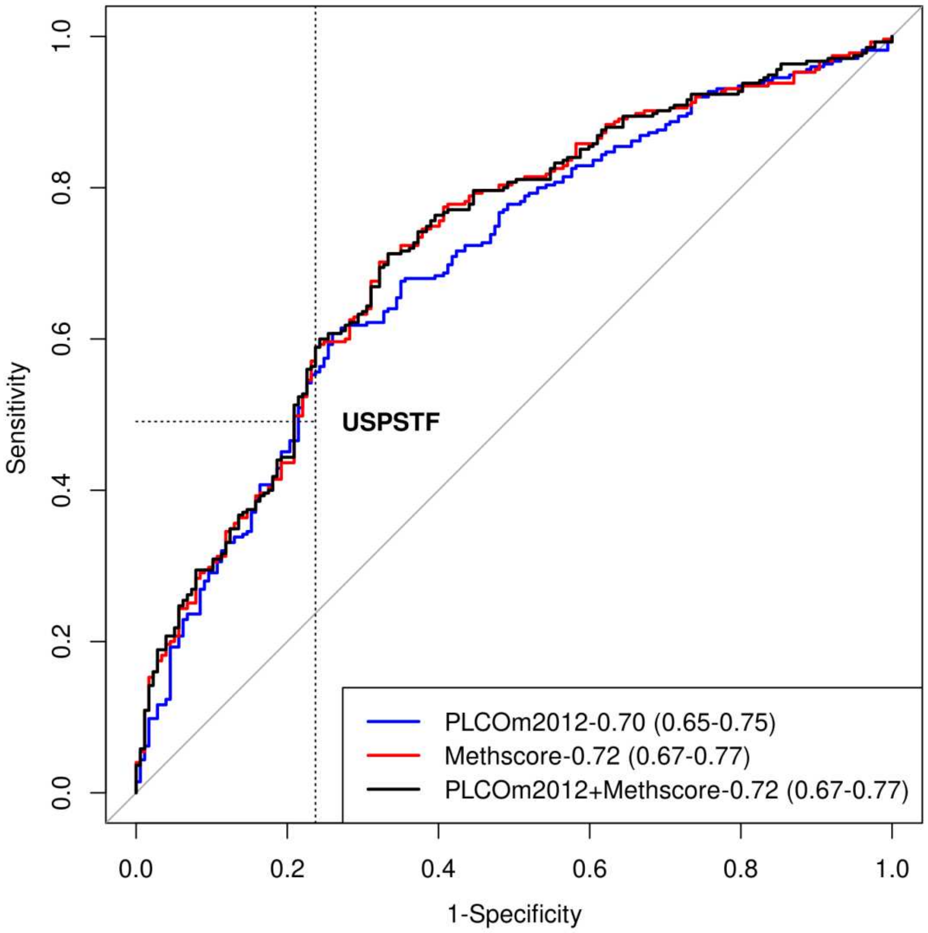

Supplement: Supplementary data [file bmjonc-2024-000334supp001.pdf]
